# Toxoplasmic Encephalitis in an AIDS Patient with Normal CD4 Count: A Case Report

**Published:** 2018

**Authors:** Eissa SOLEYMANI, Farhang BABAMAHMOODI, Lotfollah DAVOODI, Amirkeivan MAROFI, Peyman NOOSHIRVANPOUR

**Affiliations:** 1. Student Research Committee, Razi Teaching Hospital, Mazandaran University of Medical Sciences, Sari, Iran; 2. Antimicrobial Resistance Research Center, Mazandaran University of Medical Sciences, Sari, Iran; 3. Dept. of Pathology, Management of Social Security Mazandaran Province, Qaemshahr, Iran

**Keywords:** *Toxoplasma gondii*, HIV, Toxoplasmic encephalitis, Ring enhancement lesion

## Abstract

Toxoplasmic encephalitis is a common presentation of *Toxoplasma gondii* infection of the central nervous system in the late stage in AIDS patients. A 40 yr old female patient was admitted to Razi Hospital of Qaemshahr City in north of Iran, in Nov 2015, with complaint of headache, blurring of vision, dysarthria and acute left-side hemiplegia and right-side ptosis. Magnetic Resonance Imaging (MRI) was performed with intravenous contrast that showed a ring enhancement lesion in the right basal ganglia showing toxoplasmic encephalitis. Anti-*Toxoplasma* IgG was positive. HIV antibody test was positive, as well. She was treated successfully with antiparasitic and Anti-HIV drugs and eventually was discharged from hospital. *T. gondii* infection is commonly detected by serologic tests. Even if in this patient, brain imaging is essential for suitable diagnosis and supervision, its results are not pathognomonic.

## Introduction

Toxoplasmic encephalitis (TE) is caused by reactivation of latent infection by the protozoan *Toxoplasma gondii* because of progressive loss of cellular immunity. Approximately 90% of patients with TE have CD4+ T-Lymphocyte count less than 200 cells/mm^3^ and 75% have CD4+ T-Lymphocyte count less than 100 cells/mm^3^ at the time of clinical appearance. The most general signs contain fever, confusion, headache, and lethargy. Seizures develop in up to 30% of patients. 70% have focal neurologic symptom such as ataxia, sensory deficits, and hemiparesis (1).

HIV effects on the brain can be evident at any level of immune function but might develop more obviously through disease development such as toxoplasmosis. HIV infection is considered in some particular clinical syndromes, laboratory abnormalities and undesired responses to remedial interventions. Cognitive deficiency is quite common and without treatment, a large portion of patients with HIV infection would develop a clinical brain disorder. Space-occupying lesions in the brain are common in advanced the most stage of HIV. Especially primary brain lymphoma and brain abscess are due to reactivation of *T. gondii* (2).

The most finding neuro pathology in brain of TE is multifocal necrotizing encephalitis that progresses to parenchymal abscesses and surrounding inflammation (3). In every high-income settings with high seroprevalence, in the absence of prophylaxis, 30% to 40% of patients with AIDS will progress TE (4). Similarly to most CNS diseases in AIDS patient, diagnosis of TE is often difficult. In clinical practice treatment of TE, generally begins upon presumption based on clinical and radiological features as well as response to treatment (5). About 75% of patients with TE have CD4 count less than 100 cells/mm^3^ (1) such as report that patient had very low CD4 count (27 cells/mm^3^) (6). Here, we present a case of a female Iranian with HIV/AIDS and normal CD4+ count (500 cells/mm^3^) who had TE.

## Case report

**Fig. 1:**
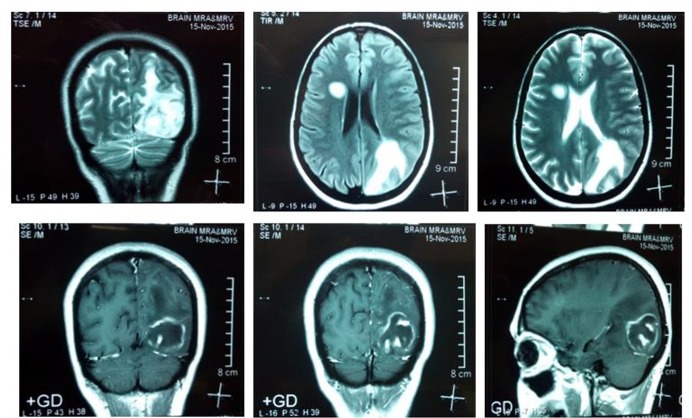
The T_1_-weighted MRI after gadolinium injection shows multiple rings enhancement lesions with surrounding edema in right temporal left parietal lobe of brain a patient with toxoplasmic encephalitis

**Fig. 2:**
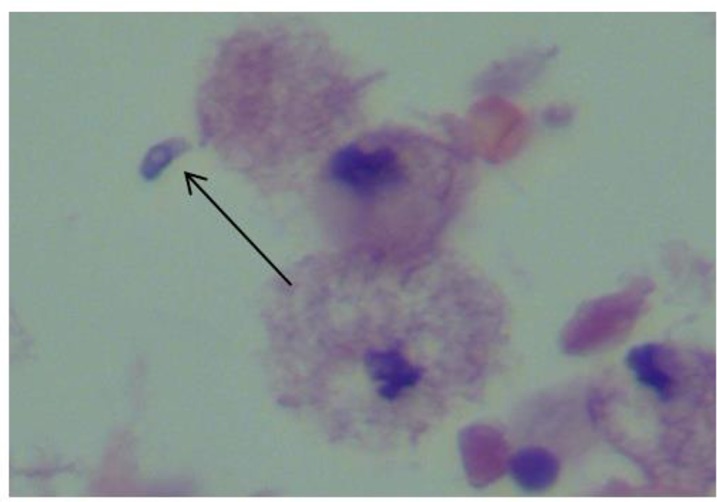
Tachyzoite in brain section smear (H&E stain, ×400) (Original)

A 40-yr-old female patient was admitted to of Razi Hospital of Qaemshahr City in north of Iran in Nov 2015 with complaint of headache, blurring of vision, dysarthria and acute left-side hemiplegia and right-sided ptosis. Three weeks ago, she had gone to another hospital that after checking she was diagnosed with brain abscess. Magnetic Resonance Imaging (MRI) with intravenous contrast was performed and showed a ring enhancement lesion in the right basal ganglia (Fig. 1). Despite the performed MRI and diagnosis of TE, biopsy of brain was done and the biopsy sample sent to pathologist. In pathology slide, tachyzoite of *T. gondii* was seen. Observations of tachyzoites show reactivation of parasites considered as indicator of TE (Fig. 2).

In lab data Anti *Toxoplasma* IgG was positive (other laboratory tests in the below table have been brought) (Table 1). HIV antibody test was requested which revealed positive by ELISA method that Western blot method confirmed it. Her husband was an addict and died a few years ago. Toxoplasmosis treatment was done with pyrimethamine, sulfadiazine, folinic acid, and dexamethasone for six weeks that decreased Anti *Toxoplasma* IgG significantly. Moreover, triple therapy of Anti-HIV drugs (Tenofovir, emtricitabine, and efavirenz) was performed. She was discharged from hospital in relatively good condition. For follow up of this patient, imaging of brain was done in which ring enhancement lesion was eliminated.

**Table 1:** Laboratory Results of a HIV patient with toxoplasma encephalitis

***Tests***		***Results***	***Unit***	***Reference value***
Withe Blood Cell		7.9×10^3^	mm^3^	4–11×10^3^
Red Blood Cell		4.26×10^6^	mm^3^	4.2–5.6×10^6^
Haemoglobin		12.4	(g/dl)	11–17
Platelets per mm^3^		256×10^3^	mm^3^	150–450×10^3^
White cell count Differentials (%)				
Neutrophils		74	(%)	40–80
Lymphocytes		18	(%)	20–40
Eosinophils		7	(%)	1–6
Monocyte		1	(%)	2–10
Erythrocyte sedimentation rate (ESR)		**43 (High)**	(mm/h)	12–32
Fasting blood sugar		96	(mg/dl)	70–105
Urea		45	(mg/dl)	12–45
Creatinine		0.8	(mg/dl)	0.5–1.3
Lactate dehydrogenase (LDH)		262	IU/I	125–450
Serum Albumin		4.2	g/dl	3.5–5
S.G.O.T		17	IU/I	5–40
S.G.P.T		16	IU/I	5–40
A.PHOSPHATASE (ALP)		158	U/I	64–306
*Toxoplasma* Ab IgG		82(High)	IU/ml	Negative:<7.2 Equivocal:7.2–8.8 Positive:>8.8
*Toxoplasma* Ab IgM		< 3	Au/ml	Negative:<6 Equivocal:6–8 Positive:>8
HIV screen (Elisa)		Positive	-	Negative
H.B.S Ag (Elisa)	-	Negative	-	Negative
H.C.V Ab (Elisa)		Negative	-	Negative
CD4		**500**	(%)	500–1500 cells/mm^3^
HIV *		Positive	-	Negative

* was confirmed by Western blot method

## Discussion

We present a case of TE in HIV infected patient in Iran through the count of CD4+ in this patient was normal. Her husband was an addict and probably before dying had transmitted HIV virus to her with sexual transmission. Clinical and paraclinical data confirmed reactivation of *T. gondii* in her brain. She lived in southwest of Iran and migrated to rural area of north of Iran and probably infected with this parasite several years ago. This infection has been as latent toxoplasmosis infection that appears as encephalitis. Typically in Iran, infection of *T. gondii* was occurred by polluted water and soil since in their eating habits undercooked or raw meat is not used nutritional habits (7). North of Iran is endemic to toxoplasmosis and maximum infection level has been reported from there because it has a moderate temperature and high moisture suitable for *T. gondii* (7–9).

TE in AIDS patients with normal CD4 count is rare. Many studies have reported a strong relationship between CD4 cell counts of less than 100/mm^3^ and the development of TE (10). We found many papers about TE in AIDS patients with normal CD4 count. TE that CD4 was less than 100 cells/μl (6, 11, 12). In Cameroon, head CT scan findings, clinical presentation, fatality rate, and median CD4 counts of 97 HIV positive patients caused to TE show that middle CD4 cell counts was 68/mm^3^ (13).TE is more common in the advanced stage of HIV disease when CD4 count is low (10) while CD4 count of our patient was sufficient. Occurrence of TE among HIV infected patients were determined 14.4% and states of severe immune deficiency with TE infection reveals latent infection of *Toxoplasma gondii* and normally described in ring enhancement lesion found in 81.4% of patients (13).

While the attendance of several ring enhancement lesions with surrounding swelling and a positive serology is extremely indicator of TE, other current central brain lesions in HIV-infected patients must be considered and these contain progressive multifocal leukoencephalopathy, tuberculosis and primary CNS lymphoma (14).

TE is a common cause of morbidity and mortality among severely immune compromised HIV infected patients. A definitive diagnosis of TE is still difficult in most centers. The fatality rate of TE was 29.9%, 31.6% and 23% in HIV infected patients, respectively (13, 15, 16). Because case fatality rate of TE is high; therefore primary prophylaxis with adequate compliance must be instituted between patients with severe immune incrassation as well as early initiation of antiretroviral therapy in HIV infected patients to avoid severe immune defect especially in endemic area such as north of Iran (17).

## Conclusion

*T. gondii* infection is commonly detected by serologic tests but in TE brain imaging is essential for suitable diagnosis and supervision. Clinical response to treatment usually is good and rapid but has delayed radiology response.
